# Correlation between ratio of fasting blood glucose to high density lipoprotein cholesterol in serum and non-alcoholic fatty liver disease in American adults: a population based analysis

**DOI:** 10.3389/fmed.2024.1428593

**Published:** 2024-09-04

**Authors:** Xianjing Jin, Jing Xu, Xiaochun Weng

**Affiliations:** ^1^Department of Ultrasound, The Second Affiliated Hospital and Yuying Children’s Hospital of Wenzhou Medical University, Wenzhou, China; ^2^Department of Ultrasound, Wenzhou Yongjia County Traditional Chinese Medicine Hospital, Wenzhou, China; ^3^Department of Endocrinology, The Second Affiliated Hospital and Yuying Children’s Hospital of Wenzhou Medical University, Wenzhou, China

**Keywords:** fasting blood glucose, fatty liver, obesity, diabetes, dyslipidemia

## Abstract

**Background:**

Based on previous research, elevated fasting blood glucose (FBG) and decreased high-density lipoprotein cholesterol (HDL-C) levels are associated with non-alcoholic fatty liver disease (NAFLD). It is hypothesized that the prevalence of NAFLD may be proportional to the FBG-to-HDL-C ratio (GHR).

**Methods:**

In this study, 3,842 participants from the National Health and Nutrition Examination Survey (NHANES) (2013–2020) were investigated. Liver steatosis was assessed using vibration-controlled transient elastography (VCTE). NAFLD was defined as controlled attenuation parameter (CAP) ≥288 dB/m.

**Results:**

After adjusting for race, gender, age, diabetes, BMI, moderate activities, uric acid, albumin, ALT, GGT, ALP, total bilirubin and creatinine, multiple logistic regression analysis indicated a positive correlation between GHR and the prevalence of NAFLD (OR = 1.22, 95% CI = 1.17–1.28). Additionally, multiple linear regression analysis showed a positive correlation between GHR and the severity of liver steatosis according to CA *p*-values (*β* = 4.97, 95% CI: 4.28, 5.66). According to the subgroup analysis, the correlation was stronger in other race, participants at the age <50 years old and those with non-diabetes. In this study, a non-linear relationship and saturation effect between GHR and the prevalence of NAFLD was also revealed, characterized by an inverted L-shaped curve, with an inflection point of 7.443. Finally, the receiver operating characteristic (ROC) analysis suggested that the area under the curve (AUC) of GHR (AUC = 0.731) significantly exceeded that of FBG and HDL-C.

**Conclusion:**

Elevated GHR levels are independently associated with the severity of liver steatosis and the increased prevalence of NAFLD in American adults.

## Introduction

The prevalence and incidence of NAFLD are increasing worldwide (1–4), with rates from 13% in Africa (1) to 42% in Southeast Asia (4). In the United States, the prevalence of NAFLD is currently 35.3% (5) and continues to increase (4). Due to the increase of prevalence, NAFLD is increasingly recognized as a significant factor to liver fibrosis, cirrhosis, liver transplantation and hepatocellular carcinoma (HCC), resulting in a substantial socioeconomic burden on society (4, 6) and a rising cause of liver-related mortality globally (3). Therefore, it is necessary to discover a cost-effective and efficient biomarker for early detection and staging of fatty liver disease (7).

NAFLD has been found to have a strong and reciprocal correlation with type 2 diabetes mellitus (T2DM), obesity, hypertension and dyslipidemia, serving as a hepatic manifestation of metabolic syndrome (MetS) (2, 8). Several studies have demonstrated the significant impact of blood glucose in serum on the initiation and advancement of NAFLD. Specifically, it has been shown that the concentrations of elevated 24 h glucose can increase hepatic *de novo* lipogenesis (DNL) in NAFLD patients (9). Furthermore, HDL-C, commonly known as “good cholesterol,” is crucial in binding lipid molecules, such as triglyceride (TG) and cholesterol, thereby actively contributing to the process of clearing cholesterol and ultimately preventing the progression of NAFLD (10). Due to the opposite trend between glucose and HDL-C, there is a greater difference in GHR between non NAFLD and NAFLD groups, making it a potential biomarker for diagnosing NAFLD. To our knowledge, it is the first study to report the effectiveness of GHR in the diagnosis of NAFLD.

In this study, the data collected from the NHANES (2013–2020) cohort were adopted to examine and evaluate the correlation between GHR and the prevalence of NAFLD in American adults.

## Materials and methods

### Research design and research population

The data analyzed in this study were obtained from NHANES (2013–2020), with a stratified, multi-stage probability and complex sample of uninstituted population in the United States. The cross-sectional surveys were conducted by NCHS.

**Figure 1:**
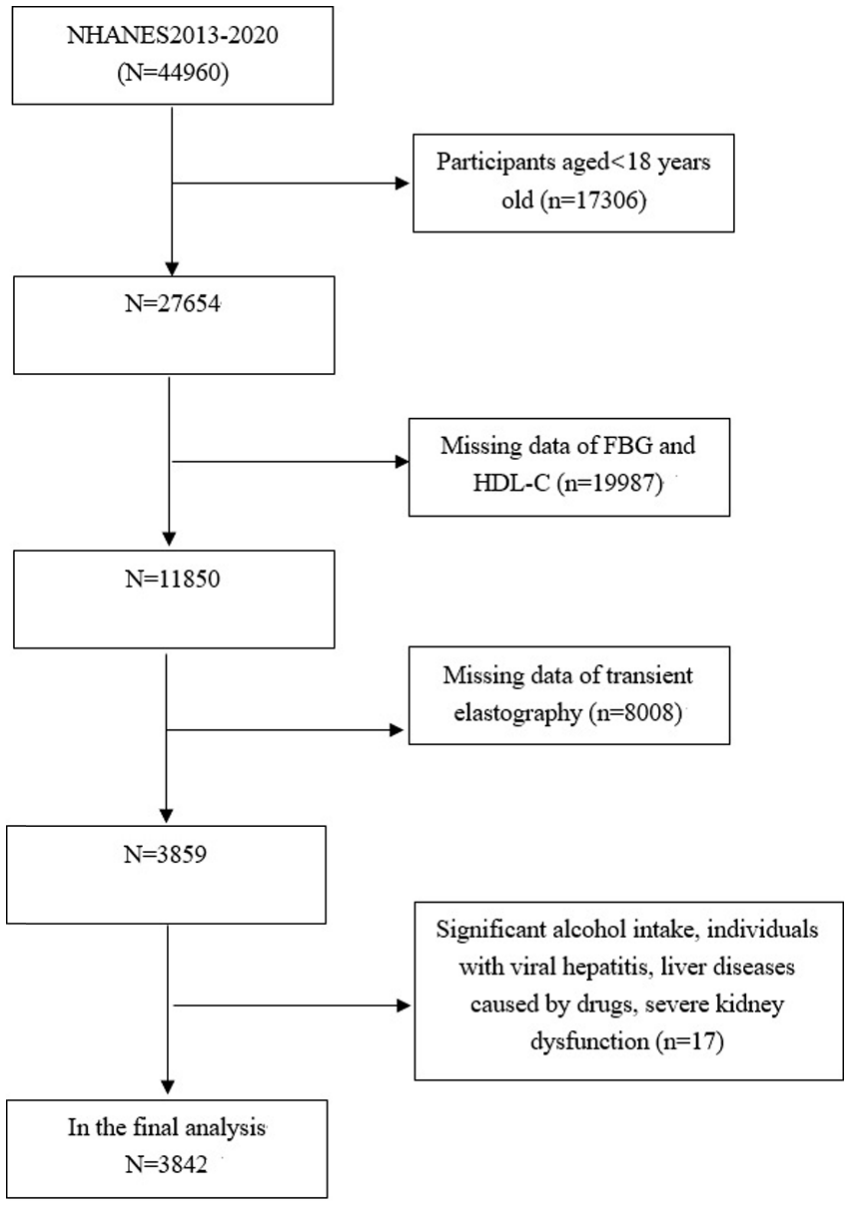
Flowchart of the sample selection from the 2013–2020 NHANES.

The study focuses exclusively on subjects aged 18 years old and above (*n* = 27,654), among which, 23,812 subjects were excluded: (1) those with missing data on FBG, HDL-C or transient elastography (TE); (2) those who self-reported high levels of alcohol consumption, defined as exceeding 14 drinks for females and 21 drinks for males weekly; (3) those with viral hepatitis, severe kidney dysfunction, liver diseases caused by drugs. Consequently, 3,842 subjects aged 18–80 years old were included in the final analysis (Figure 1).

The implementation of NHANES was approved by the Ethics Review Board of NCHS, and all subjects have provided the informed consent in written (11).

### Vibration controlled transient elastography

In the database of NHANES (2013–2020), liver vibration controlled transient elastography (VCTE) in participants was measured with the FibroScan 502 V2 Touch (Echosens), which was well-suited for studying NAFLD, a condition characterized by fatty liver disease. In order to assess liver steatosis in patients with fatty liver disease, validated parameters such as controlled attenuation parameter (CAP) were adopted (12, 13). The VCTE results were considered effectively to follow certain criteria, including obtaining at least 10 LSMs after fasting for at least 3 h, and interquartile range (IQR)/median less than 30% (14). It is used to determine that CAP values (≥288 dB/m) were NAFLD status (15).

### Research variables

The following covariates, including ALT, age, BMI, gender, history of diabetes, race [non-Hispanic White, non-Hispanic Black, Hispanic (American Mexican, and Hispanic other), and other race/multiracial], moderate activities, weight, TC, albumin, GGT, creatinine, ALP, LDL-C, FBG, HDL-C, total bilirubin, and triglyceride (TG) were included. Details of NAFLD and other covariate acquisition process were available at www.cdc.gov/nchs/nhanes/.

### Statistical analysis

GHR was determined by calculating the ratio of FBG (mmol/L) to HDL-C (mmol/L). Continuous data in this analysis were presented as weighted mean ± standard deviation (SD). Categorical variables were represented as weighted proportions, and subjects were stratified into quartiles based on their GHR levels. The differences among the groups for categorical variables were assessed with the weighted *χ*^2^ test, while a weighted linear regression model was employed for continuous variables. Furthermore, the relationship between GHR and NAFLD status was examined with a weighted multivariate logistic regression model. In addition, the correlation between GHR and liver steatosis was explored through a weighted multivariate linear regression analysis, and assessed by liver CAP. The potential impacts of gender, BMI, age, moderate activities, diabetes and race on the relationship between GHR and NAFLD were examined through subgroup analyses. To identify any potential non-linear relationships between GHR and NAFLD probabilities, smooth curving fits and generalized additive models were utilized. The AUC was calculated through ROC analysis to evaluate the diagnostic ability of GHR, FBG and HDL-C for identification of NAFLD. Moreover, the statistical significance of the difference between two AUC values was assessed by MedCalc version 12.1.4.0 (MedCalc software, Belgium). Statistical analyses were performed with EmpowerStats software and R, with a significance (*p* < 0.05).

## Results

### Baseline characteristics of participants

A total of 3,842 participants aged 18–80 years old were included in the study, with a prevalence of NAFLD of 35.2%. The distribution of participant characteristics stratified by serum GHR quartiles (Q1: <3.49; Q2: 3.49–4.42; Q3: 4.42–5.66; Q4: >5.66) has been presented in Table 1. Compared with the bottom quartile, those in the top quartile of GHR were more likely to be the elderly and males, with a higher proportion of Mexican Americans, a higher prevalence of NAFLD, diabetes, and the increased levels of ALT, weight, GGT, BMI, ALP, creatinine, FBG, uric acid, TG, and CAP. In contrast, the proportion of moderate activities, and the levels of albumin, TC, HDL-C were lower (*p* < 0.05).

**Table 1:** Weighted characteristics of the study population based on GHR quartiles.

Characteristic	Q1	Q2	Q3	Q4	*p*-value
Number	960	961	963	958	
Age, year	48.5 ± 18.4	47.1 ± 18.5	48.8 ± 17.9	53.1 ± 16.4	<0.001
NAFLD, %					<0.001
Yes	14.2	23.8	36.8	62.4	
No	85.8	76.2	63.2	37.6	
Sex, %					<0.001
Male	27.4	43.5	56.8	65.7	
Female	72.6	56.5	43.2	34.3	
Race, %					<0.001
Mexican American	9.5	14.2	15.5	17.9	
Other Hispanic	8.8	9.0	12.0	11.9	
Non-Hispanic White	37.8	32.4	37.1	37.1	
Non-Hispanic Black	26.7	25.8	19.8	17.1	
Other Race	17.2	18.5	15.6	16.0	
Moderate activities, %					<0.001
Yes	47.6	41.2	38.5	34.5	
No	52.4	58.8	61.5	65.5	
Diabetes					<0.001
Yes	3.8	5.7	10.6	38.1	
No	96.2	94.3	89.4	61.9	
Weight, kg	71.4 ± 17.7	79.8 ± 21.3	86.9 ± 21.3	94.6 ± 24.2	<0.001
BMI, kg/m^2^	26.2 ± 5.9	28.8 ± 7.5	30.8 ± 7.1	33.2 ± 7.8	<0.001
GHR	2.89 ± 0.45	3.95 ± 0.27	5.00 ± 0.35	8.12 ± 3.41	<0.001
Albumin, g/dL	4.05 ± 0.34	4.04 ± 0.33	4.03 ± 0.33	3.99 ± 0.33	<0.001
ALT, U/L	19.5 ± 28.7	19.1 ± 11.5	22.8 ± 15.4	28.0 ± 21.5	<0.001
GGT, IU/L	29.2 ± 48.9	29.5 ± 83.1	29.9 ± 29.2	39.4 ± 47.9	<0.001
ALP, IU/L	72.7 ± 25.2	76.5 ± 22.9	78.7 ± 23.5	84.3 ± 29.8	<0.001
Total bilirubin, μmol/L	8.6 ± 5.0	8.2 ± 5.0	8.4 ± 4.7	8.4 ± 5.0	<0.001
Creatinine, mmol/L	74.1 ± 26.2	77.1 ± 36.6	80.7 ± 43.0	82.2 ± 47.0	<0.001
Uric acid, μmol/L	290.9 ± 77.7	313.3 ± 80.3	340.7 ± 80.4	353.8 ± 91.0	<0.001
FPG, mmol/L	5.3 ± 0.5	5.6 ± 0.6	6.0 ± 0.8	8.2 ± 3.3	<0.001
TC, mmol/L, mmol/L	4.98 ± 0.98	4.76 ± 1.03	4.69 ± 1.03	4.54 ± 1.12	<0.001
TG, mmol/L	0.94 ± 0.42	1.16 ± 0.58	1.44 ± 0.73	2.05 ± 1.84	<0.001
LDL-C, mmol/L	2.72 ± 0.86	2.86 ± 0.90	2.90 ± 0.90	2.71 ± 0.97	0.059
HDL-C, mmol/L	1.89 ± 0.38	1.43 ± 0.16	1.20 ± 0.17	1.02 ± 0.21	<0.001
CAP, dB/m	231.2 ± 54.3	249.3 ± 55.8	271.6 ± 58.1	304.4 ± 58.7	<0.001

Values are mean ± SD or number (%). *p* < 0.05 was deemed significant. BMI, body mass index; AC, arm circumference; HbA1c, glycosylated hemoglobin; TC, total cholesterol; TG, triglyceride; HDL-c, high density lipoprotein cholesterol; LDL-c, low density lipoprotein cholesterol; ALT, alanine aminotransferase; GGT, gamma-glutamyl transpeptidase; ALP, alkaline phosphatase; LSM, liver stiffness measurements; CAP, controlled attenuation parameter.

### Correlation between GHR and the risk of NAFLD

Three weighted multivariate regression models were constructed to test the relationship between the prevalence of NAFLD and GHR (Table 2). The unadjusted model revealed a positive correlation between the levels of GHR and the probabilities of NAFLD [OR = 1.44, 95% CI: (1.38, 1.50)]. After adjusting for race, gender, age (Model 2), diabetes, BMI, moderate activities, uric acid, albumin, ALT, GGT, ALP, total bilirubin and creatinine (Model 3), the positive correlation was remained in Model 2 [OR = 1.41, 95% CI: (1.35, 1.47)] and Model 3 [OR = 1.22, 95% CI: (1.17, 1.28)]. Moreover, compared with the lowest level of GHR (Q1) in Model 3 (*p* for trend <0.001), the risk of NAFLD in subjects in quartiles 2, 3 and 4 increased by 0.35, 0.96 and 2.72, respectively. This results indicate that adults with elevated GHR are more likely to develop NAFLD than those with reduced GHR.

**Table 2:** Association between GHR and NAFLD status in logistic regression analysis.

	Model 1 OR (95% CI) *p*-value	Model 2 OR (95% CI) *p*-value	Model 3 OR (95% CI) *p*-value
GHR	1.44 (1.38, 1.50), <0.001	1.41 (1.35, 1.47), <0.001	1.22 (1.17, 1.28), <0.001
AC (Quartile)			
Q1	Reference	Reference	Reference
Q2	1.87 (1.48, 2.35), <0.001	1.90 (1.51, 2.40), <0.001	1.35 (1.05, 1.75), 0.022
Q3	3.55 (2.85, 4.42), <0.001	3.55 (2.83, 4.45), <0.001	1.96 (1.52, 2.52), <0.001
Q4	9.41 (7.55, 11.73), <0.001	8.95 (7.12, 11.24), <0.001	3.72 (2.86, 4.84), <0.001
*p* for trend	<0.001	<0.001	<0.001

Model 1: None covariates were adjusted. Model 2: Gender, age and race were adjusted. Model 3: Gender, age, race, diabetes, moderate activities, BMI, albumin, uric acid, ALT, GGT, ALP, total bilirubin, creatinine.

### Correlation between GHR and the severity of liver steatosis

A multivariate linear regression analysis was performed between CAP and GHR (Table 3). GHR in Model 3 was dramatically and positively correlated with the severity of liver steatosis according to CAP values (*β* = 4.97, 95% CI: 4.28, 5.66) (*p* < 0.001).

**Table 3:** Associations between GHR and CAP value in linear regression analysis.

	Model 1 *β* (95% CI) *p*-value	Model 2 *β* (95% CI) *p*-value	Model 3 *β* (95% CI) *p*-value
GHR	9.31 (8.60, 10.01), <0.001	8.60 (7.89, 9.32), <0.001	4.97 (4.28, 5.66), <0.001
GHR (Quartile)			
Q1	Reference	Reference	Reference
Q2	18.10 (13.02, 23.18), 0.001	18.69 (13.66, 23.71), <0.001	7.44 (2.97, 11.91), <0.001
Q3	40.45 (35.38, 45.53), <0.001	39.85 (34.75, 44.95), <0.001	18.19 (13.51, 22.87), <0.001
Q4	73.18 (68.09, 78.26), <0.001	70.01 (64.82, 75.20), <0.001	36.79 (31.69, 41.89), <0.001
*p* for trend	<0.001	<0.001	<0.001

Model 1: None covariates were adjusted. Model 2: Gender, age and race were adjusted. Model 3: Gender, age, race, diabetes, moderate activities, BMI, albumin, uric acid, ALT, GGT, ALP, total bilirubin, creatinine.

### Subgroup analysis

The consistency of the correlation between GHR and the prevalence of NAFLD across different demographic variables was evaluated through subgroup analyses. As displayed in Table 4, the results indicate that the positive correlation between GHR and the risk of NAFLD remains consistent regardless of BMI, gender, and moderate activities (*p* > 0.05 for all). The correlation between GHR and the risk of NAFLD was stronger among other race (OR = 1.42, *p* interaction =0.011), participants with age <50 years old (OR = 1.38, *p* interaction <0.001), and those with non-diabetes (OR = 1.36, *p* interaction <0.001).

**Table 4:** Association between GHR and NAFLD stratified by gender, age, race, diabetes, moderate activities and BMI.

	OR (95% CI) *p*-value	*p* for interaction
Stratified by gender		0.204
Male	1.17 (1.11–1.23), <0.001	
Female	1.29 (1.20–1.38), <0.001	
Stratified by race		0.011
Mexican American	1.24 (1.10, 1.39), <0.001	
Other Hispanic	1.16 (1.04, 1.29), 0.009	
Non-Hispanic White	1.25 (1.15, 1.37), <0.001	
Non-Hispanic Black	1.10 (1.02, 1.19), 0.016	
Other Race	1.42 (1.26, 1.60), <0.001	
Stratified by age		<0.001
Age <50 years old	1.38 (1.27, 1.50), <0.001	
Age ≥50 years old	1.17 (1.11, 1.22), <0.001	
Stratified by BMI		0.139
BMI <30 kg/m^2^	1.31 (1.23, 1.39), <0.001	
BMI ≥30 kg/m^2^	1.19 (1.12, 1.26), <0.001	
Stratified by diabetes		<0.001
Non-diabetes	1.36 (1.27, 1.46), <0.001	
Diabetes	1.09 (1.03, 1.16), 0.002	
Stratified by moderate activities		0.126
No	1.20 (1.14, 1.27), <0.001	
Yes	1.27 (1.18, 1.37), <0.001	

Gender, age, BMI, race, moderate activities, diabetes (not adjusted for in the subgroup analyses), albumin, uric acid, ALT, GGT, ALP, total bilirubin, creatinine were adjusted.

### Non-linearity and threshold effect analysis between GHR and NAFLD

**Figure 2:**
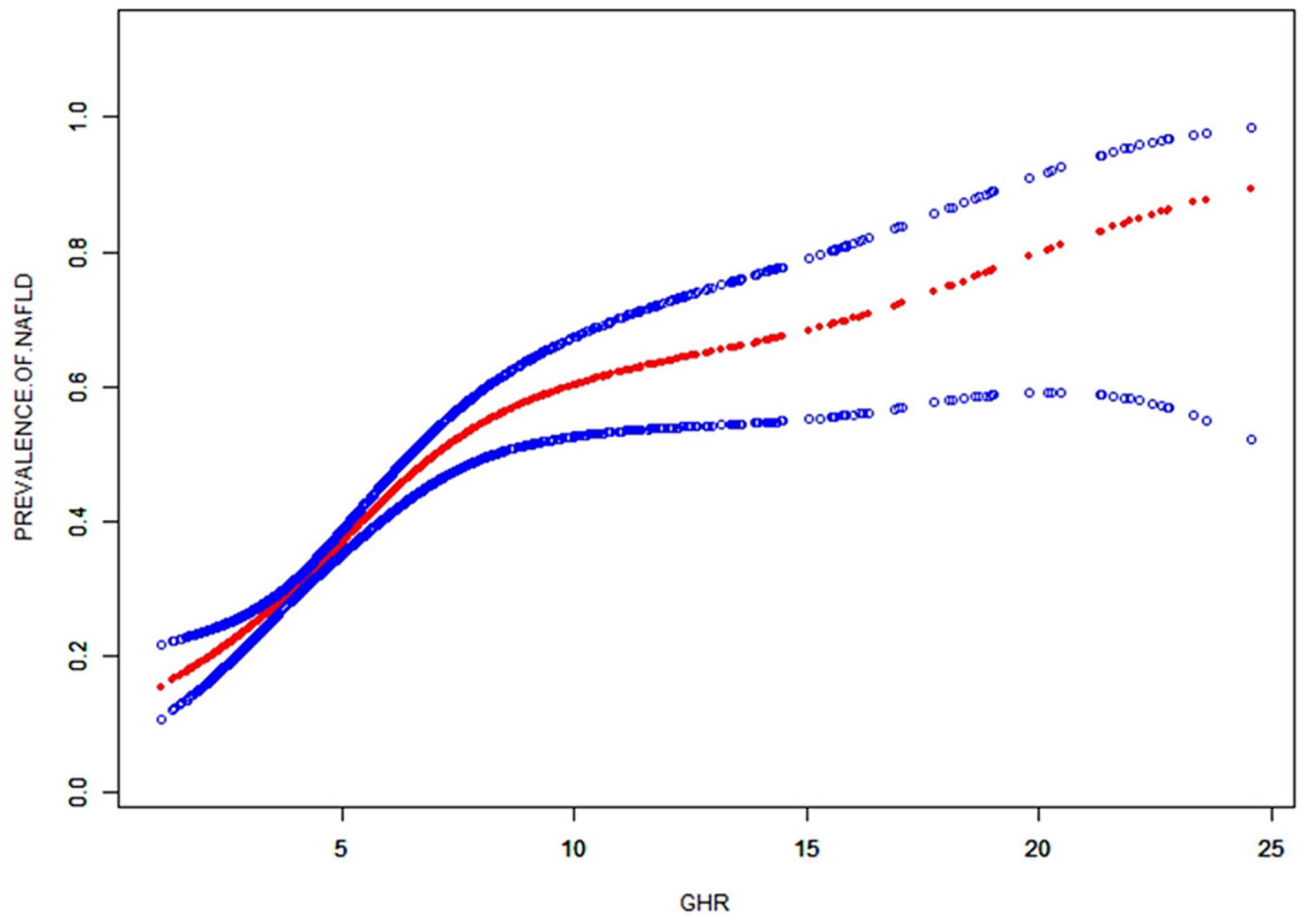
The smooth curve fit for the association between GHR and prevalence of NAFLD. Solid redline represents the smooth curve fit between variables. Blue bands represent the 95% of confidence interval from the fit. Adjusted for: race, gender, age, diabetes, BMI, moderate activities, uric acid, albumin, ALT, GGT, ALP, total bilirubin and creatinine.

**Figure 3:**
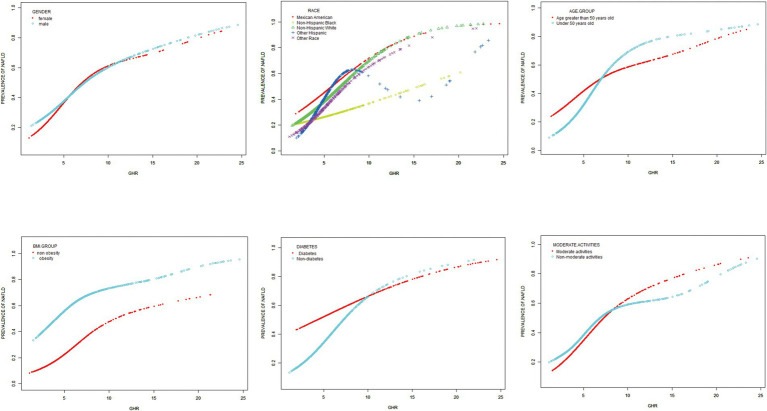
Subgroups analysis for the association between GHR and prevalence of NAFLD by gender, BMI, race, age, diabetes, moderate activities. Adjusted for: race, gender, age, diabetes, BMI, moderate activities, uric acid, albumin, ALT, GGT, ALP, total bilirubin and creatinine.

A generalized additive model and smooth curve fittings were employed to illustrate the non-linear relationship and saturation effect between GHR and NAFLD, as depicted in Figures 2, 3. Among the participants, the correlation between GHR and NAFLD displayed an inverted L-shaped curve, with inflection points of 7.443 (as Table 5). Below the threshold of 7.443, a significant effect value of 1.359 was observed, while the value dropped to 1.076 when GHR exceeded 7.443.

**Table 5:** Threshold effect analysis of GHR on NAFLD using the two-piecewise linear regression model.

GHR	Adjusted OR (95% CI) *p*-value
Fitting by the standard linear model	1.218 (1.164, 1.274), <0.001
Fitting by the two-piecewise linear model	
Inflection point	7.443
GHR <7.443	1.359 (1.267, 1.457), <0.001
GHR >7.443	1.076 (1.005, 1.153), 0.0348
Log likelihood ratio	<0.001

### ROC analysis

**Figure 4:**
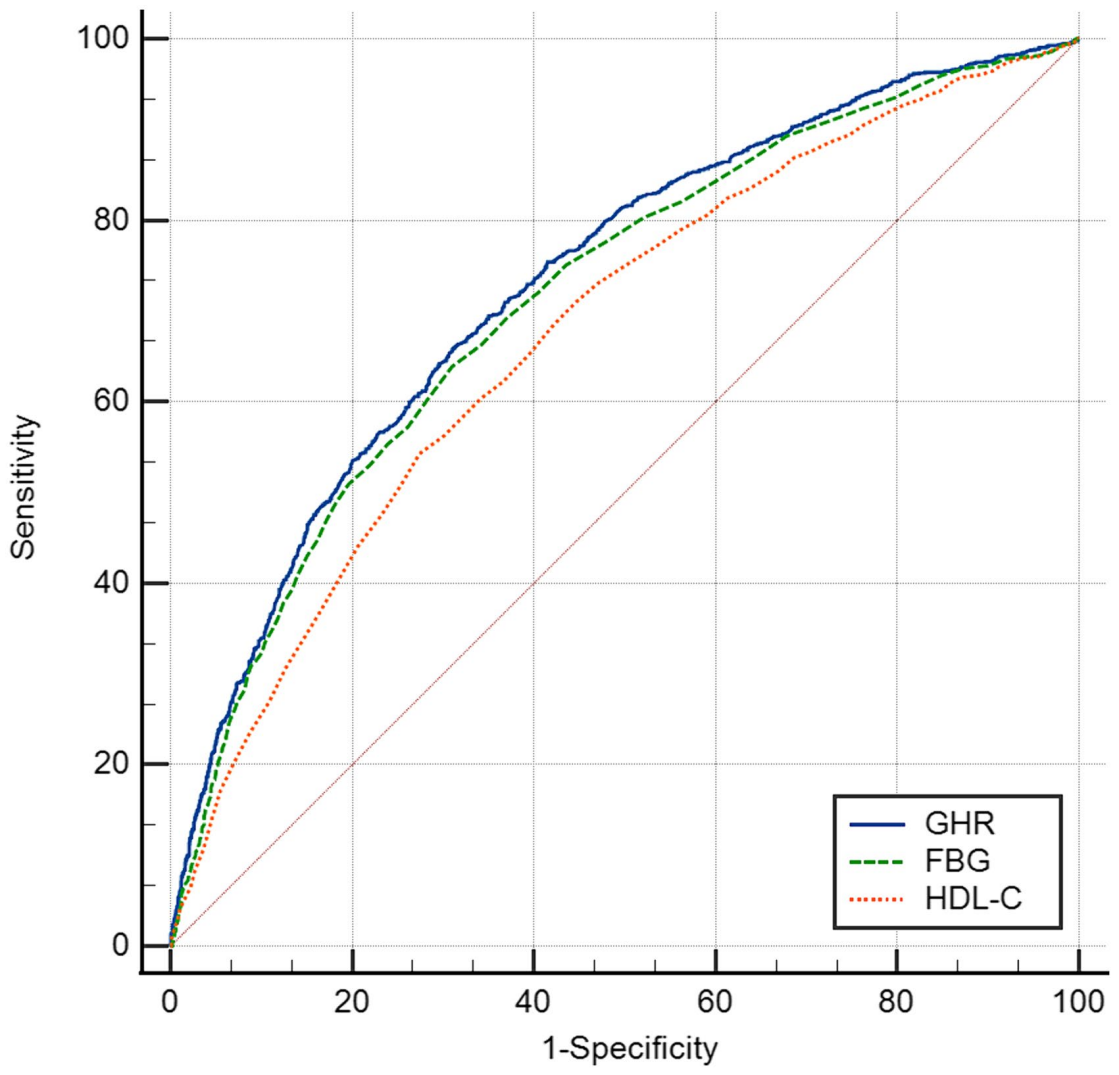
Receiver operating characteristic curves of GHR to identify NAFLD.

The ROC in Figure 4 and Table 6 presents the diagnostic performance of GHR, FBG and HDL-C in identifying NAFLD. The AUC for GHR in the ROC analysis was notably higher than that of FBG and HDL-C at 0.731 (95% CI: 0.714–0.747), with a sensitivity of 66.0%, a specificity of 68.8% and a cutoff of 4.73.

**Table 6:** The AUC for each index to discriminate NAFLD.

	AUC	95% CI	Cutoff value	Sensitivity	Specificity
GHR^a^^,^^b^	0.731	0.714–0.747	4.727	0.660	0.688
FBG^b^	0.715	0.698–0.732	5.855	0.639	0.690
HDL-C	0.676	0.658–0.694	1.205	0.659	0.688

a Indicates a significant difference as compared to FBG.

b Indicates a significant difference as compared to HDL-C.

## Discussion

An elevated GHR demonstrated a significant correlation with the prevalence of NAFLD in a large adult population in the United States in this cross-sectional study. Through subgroup analyses and interaction assessment, a stronger correlation was discovered in other race, participants with age <50 years old and those with non-diabetes. The analysis revealed an inverted L-shaped relationship between GHR and the prevalence of NAFLD, with a notable inflection point at a GHR measurement of 7.443. Furthermore, GHR exhibited a superior diagnostic accuracy for NAFLD compared to FBG and HDL-C alone.

Numerous studies have shown that glucose stimulates hepatic *de novo* lipogenesis (DNL) by activating carbohydrate-responsive element-binding protein (ChREBP) (16, 17). Moreover, studies have shown that fluctuations in glucose levels contribute to hepatic apoptosis, fibrosis, and inflammation by increasing oxidative stress in both *in vitro* and *in vivo* (18, 19). Conversely, HDL-C has been found to inhibit the retention, buildup, and oxidation of LDL-C, thereby exerting a protective effect. HDL-C facilitates the removal of dietary cholesterol through the reverse cholesterol transport pathway and exhibits antioxidant and anti-inflammatory properties (20). Therefore, a decrease in HDL-C levels may lead to impaired cholesterol efflux and antioxidant function, potentially contributing to the development of NAFLD (21). Studies have demonstrated a strong correlation between low HDL-C levels and the severity and progression of NAFLD (22, 23). The combination of HDL-C with other biomarkers has shown promising predictive value for NAFLD, with the monocyte-to-HDL-C ratio and uric acid-to-HDL-C ratio identified as independent predictors of the risk of NAFLD and severity (24, 25). Additionally, previous studies have indicated a significant correlation between the sdLDL-to-HDL-C ratio and NAFLD (26). Guo et al. (27) posited that elevated GHR levels were significantly correlated with heightened all-cause mortality among non-diabetic individuals with coronary artery disease undergoing percutaneous coronary intervention. So far, it is the first study to assess the relationship between GHR and NAFLD. The study revealed a significant correlation between elevated GHR levels and an increased prevalence of NAFLD in American adults, suggesting the potential importance of further exploration into the role of GHR in health outcomes.

Analyses on ROC revealed that GHR demonstrated the superior predictive utility for NAFLD compared to single biomarkers such as FBG and HDL-C. GHR may serve as a more effective indicator for clinical adjunct diagnosis of NAFLD due to the divergent trends observed in FBG and the levels of HDL-C. Additional research was warranted to investigate potential correlations between GHR and other metabolic conditions, such as hypertension, insulin resistance, and cardiovascular risk.

The findings suggest a notably stronger correlation between GHR and the prevalence of NAFLD among individuals under 50 years old and those with non-diabetes. As individuals ageing, body composition, metabolism, and the presence of coexisting diseases undergo changes (28–30). Additionally, dietary irregularities and insufficient exercise in young individuals can lead to excessive fat accumulation, potentially influencing GHR (31). Importantly, NAFLD is often overlooked for the aforementioned population. Therefore, GHR should be considered as an important factor for identifying NAFLD, especially for the aforementioned population.

Additionally, the study revealed an inverted L-shaped correlation between GHR and NAFLD, with the inflection point of 7.443. The discrepancy in the correlation between GHR and NAFLD on either side of the inflection point may be attributed to the influence of other variables. Analysis on Supplementary Table S1 indicated that individuals with GHR ≥7.443 exhibited higher levels or proportions than those with GHR <7.443 in male gender, BMI, weight, FBG, ALT, GGT, ALP, creatinine, uric acid and TG. However, abnormalities in these indicators were closely linked to NAFLD (32–34). When the level of GHR exceeded 7.443, the impact of GHR on NAFLD was found to be relatively weak, likely due to the presence of other risk factors for NAFLD. The study underscores the importance of targeting GHR levels in clinical interventions aimed at preventing NAFLD, with a particular emphasis on maintaining GHR levels below 7.443. Lower GHR levels below the threshold may significantly reduce the risk of NAFLD. This inflection point can serve as novel evidence supporting the management of GHR.

Notably, the study benefits from a large sample size and the national representativeness of Americans. In addition, various indicators in the model were adjusted to enhance the reliability of the findings. Nonetheless, this study is subject to certain limitations. Firstly, the establishment of a causal relationship between GHR and NAFLD was not feasible through cross-sectional studies. Secondly, the diagnosis of NAFLD relied on CAP values rather than the gold standard liver biopsy. Thirdly, the study was restricted to American adults, necessitating further prospective cohort research to validate and generalize the present results in a broader population.

## Conclusion

In summary, elevated GHR was found to be independently associated with an increased risk of NAFLD and the severity of liver steatosis in a sizable cohort of American adults. This correlation was particularly pronounced among individuals of other races, those under 50 years old, and those without diabetes. These findings underscore the potential utility of GHR as a biomarker for identifying individuals at the heightened risk for NAFLD, thereby facilitating early detection and intervention strategies for this common liver disorder.

## Data Availability

Publicly available datasets were analyzed in this study. This data can be found here: NHANES, www.cdc.gov/nchs/NHANEs/.
