# Correction: Correlation between ratio of fasting blood glucose to high density lipoprotein cholesterol in serum and non-alcoholic fatty liver disease in American adults: a population based analysis

**DOI:** 10.3389/fmed.2025.1683822

**Published:** 2025-08-22

**Authors:** Xianjing Jin, Jing Xu, Xiaochun Weng

**Affiliations:** ^1^Department of Ultrasound, The Second Affiliated Hospital and Yuying Children's Hospital of Wenzhou Medical University, Wenzhou, China; ^2^Department of Ultrasound, Wenzhou Yongjia County Traditional Chinese Medicine Hospital, Wenzhou, China; ^3^Department of Endocrinology, The Second Affiliated Hospital and Yuying Children's Hospital of Wenzhou Medical University, Wenzhou, China

**Keywords:** fasting blood glucose, fatty liver, obesity, diabetes, dyslipidemia

In the published article, there was an error in affiliation 3. Instead of “Xianjing Jin^1^, Jing Xu^2^ and Xiaochun Weng 3^*^

1 Department of Ultrasound, Wenzhou Yongjia County Traditional Chinese Medicine Hospital, Wenzhou, China.

2 Department of Endocrinology, The Second Affiliated Hospital and Yuying Children's Hospital of Wenzhou Medical University, Wenzhou, China.

3 Department of Ultrasound, The Second Affiliated Hospital and Yuying Children's Hospital of Wenzhou Medical University, Wenzhou, China.”,

it should be “Xianjing Jin^2^, Jing Xu^3^ and Xiaochun Weng^1*^

1 Department of Ultrasound, The Second Affiliated Hospital and Yuying Children's Hospital of Wenzhou Medical University, Wenzhou, China

2 Department of Ultrasound, Wenzhou Yongjia County Traditional Chinese Medicine Hospital, Wenzhou, China

3 Department of Endocrinology, The Second Affiliated Hospital and Yuying Children's Hospital of Wenzhou Medical University, Wenzhou, China”

The original article has been updated.

