# Supplementary material for: Correlation between ratio of fasting blood glucose to high density lipoprotein cholesterol in serum and non-alcoholic fatty liver disease in American adults: a population based analysis
Source: Front Med (Lausanne). 2024 Sep 4;11:1428593. doi: 10.3389/fmed.2024.1428593 (PMC11408235; doi:10.3389/fmed.2024.1428593)
Supplement: Supplementary file 1 [file Table_1.DOCX]

Table S1 The baseline characteristics of participants on both sides of the inflection point according to NAFLD.

| GHR | <7.443 | ≥7.443 | P-value |
| --- | --- | --- | --- |
| Participants | 3480 | 359 |  |
| Age, year | 48.7±18.1 | 56.1±14.5 | <0.001 |
| Male, % | 47.7 | 65.5 | <0.001 |
| Body mass index, kg/m^2^ | 29.3±7.4 | 33.7±7.6 | <0.001 |
| Weight, kg | 81.8±22.4 | 96.3±24.2 | <0.001 |
| Diabetes, % | 10.2 | 64.3 | <0.001 |
| FBG, mmol/L | 5.8±0.9 | 10.6±3.9 | <0.001 |
| ALT, U/L | 21.7±20.6 | 28.6±20.5 | <0.001 |
| GGT, U/L | 30.6±55.8 | 45.4±55.2 | <0.001 |
| ALP, U/L | 76.8±24.4 | 89.7±35.0 | <0.001 |
| Albumin, g/dl | 40.4±3.3 | 39.4±3.6 | <0.001 |
| Creatinine, umol/L | 77.7±36.1 | 86.0±60.4 | <0.001 |
| Uric acid, umol/L | 322.7±84.4 | 343.6±98.1 | <0.001 |
| Total cholesterol, mmol/L | 4.76±1.03 | 4.56±1.21 | <0.001 |
| Triglycerides, mmol/L | 1.28±0.71 | 2.49±2.71 | <0.001 |
| HDL-cholesterol, mmol/L | 1.43±0.40 | 0.99±0.23 | <0.001 |
| LDL-cholesterol, mmol/L | 2.81±0.90 | 2.63±1.00 | <0.001 |
